# Post-COVID-19 Multisystem Inflammatory Syndrome in Children and Adults: What Happens After Discharge?

**DOI:** 10.7759/cureus.24438

**Published:** 2022-04-24

**Authors:** Michael Loncharich, Seth Klusewitz, Olcay Jones

**Affiliations:** 1 Rheumatology, Walter Reed National Military Medical Center, Bethesda, USA; 2 Cardiology, Walter Reed National Military Medical Center, Bethesda, USA; 3 Pediatric Rheumatology, Walter Reed National Military Medical Center, Bethesda, USA

**Keywords:** multisystem inflammatory syndrome in adults [mis-a], multi-system inflammatory disease in children (mis-c), immunology covid 19, mis-a, mis-c, covid-19

## Abstract

Introduction

Classification criteria and practice guidelines for inpatient management of multisystem inflammatory syndrome (MIS) exist, but reports on outpatient management and clinical outcomes are lacking. Here we describe the management and clinical outcomes of four children and four adults with MIS seen at Walter Reed National Military Medical Center (WRNMMC) from diagnosis to six months follow-up.

Methods

This retrospective, case-series describes the initial presentation and management of MIS in four children and four adults seen at WRNMMC from March 2020 to September 2021. Data on each patient was collected from the time of exposure to the SARS-CoV-2 virus to six months post-diagnosis with MIS. Extracted data includes: demographics, comorbidities, initial MIS presentation, inpatient treatment, outpatient treatment, and clinical outcomes.

Results

A total of 62.5% of patients presented in shock. All pediatric patients received IVIG, methylprednisolone, and anakinra; no adult received this combination. Steroids and immunomodulatory medications were discontinued in 1-2 months outpatient. Three children and two adults had full symptomatic resolution. One child and two adults had persistent deconditioning at six months follow-up. One adult had persistent dyspnea.

Conclusions

MIS appears to be monophasic with no recurrences at six months follow-up in our patients who only required 1-2 months of glucocorticoid or immunomodulatory medications. The better outcomes in children raise the question of how much of this difference can be attributed to early combination therapy versus physiologic differences in children and adults.

## Introduction

Multisystem inflammatory syndrome (MIS) was first reported in April 2020 in children with features overlapping with Kawasaki’s disease including fever, rash, conjunctivitis, and gastrointestinal manifestations; some cases progressed to shock [1]. In May 2020, the World Health Organization (WHO) and Centers for Disease Control and Prevention (CDC) published classification criteria [2,3]. By June 2020, reports of adults with cardiovascular, dermatologic, gastrointestinal, and neurologic sequelae after SARS-CoV-2 infection emerged [4]. In July 2020, the American College of Rheumatology released practice guidelines for MIS in children (MIS-C) [5]. Subsequently, numerous papers detailed common manifestations of MIS, evaluation recommendations, and inpatient treatment algorithms.

Considering adults, CDC working criteria for MIS in adults (MIS-A) were not released until October 2020 [6]. The Brighton Collaboration published joint MIS-C/MIS-A practice guidelines in May 2021 [7]. Before that, clinicians had to extrapolate MIS-C guidelines to adults or rely on case series for guidance. In fact, the Infectious Diseases Society of America (IDSA) and CDC still reference MIS-C management guidelines for treating MIS-A [8].

There remains a paucity of data reported on post-hospitalization clinical outcomes and management. To our knowledge, only one study of MIS-C in 46 patients in the United Kingdom [9] and one case series of four adults with MIS-A in Sweden [10] report such data. Here we present these outcomes from four pediatric and four adult patients with MIS, compare differences in children versus adults, and review the literature.

## Materials and methods

Study design

This was a retrospective case series of four children meeting CDC or WHO criteria for MIS-C and four adults meeting CDC criteria for MIS-A seen at Walter Reed National Military Medical Center between March 2020 and June 2021. Cases that were suspicious for MIS but failed to meet classification criteria were excluded. Exposure to the SARS-CoV-2 virus was confirmed by RT-PCR of nasopharyngeal samples or positive serology. The Walter Reed Department of Research Programs institutional review board approved this study (WRNMMC-EDO-2021-0787).

Data collection

Clinical data was extracted retrospectively from the initial diagnosis of COVID-19 to their most recent outpatient appointment. Extracted data includes: demographics, comorbidities, clinical presentation, inpatient treatment, outpatient treatment, and outcomes at follow-up. Presentation metrics included laboratory findings, radiographic imaging, organ system involvement, and clinical symptoms. Outcomes of interest were persistence or resolution of symptoms, lab abnormalities, and radiographic findings. Frequency of follow-up varied across patients. For consistency, data was collected at fixed time points: initial diagnosis, one-month follow-up, three-month follow-up, and six-month follow-up.

Statistical analysis

Simple descriptive statistics were used to report patient demographics, frequency of clinical manifestations of MIS, and rates of clinical improvement.

## Results

Case presentations

Case 1

A 17-month-old black female with fever and reduced feeding tested positive for SARS-CoV-2 by reverse transcription-polymerase chain reaction (RT-PCR) and was managed with supportive care. One week later, she developed lethargy, reduced feeding, and fever prompting medical evaluation. She was found to be hypotensive and hypoxic requiring intubation and pressors. Lab testing revealed markedly elevated brain natriuretic peptide (BNP) and inflammatory markers. Transthoracic echocardiography (TTE) revealed diastolic dysfunction and severe mitral regurgitation (MR) but normal coronary arteries. She was treated with IVIG, anakinra, pulse-dose steroids, and aspirin. Aspirin was stopped on discharge, steroids were tapered over five weeks, and anakinra was stopped at one month follow-up because the patient was asymptomatic. Repeat TTE at three months follow-up showed normalization of diastolic function and MR. The patient remained asymptomatic at three and six months follow-up.

Case 2

An 11-year-old white male with fever, conjunctivitis, rash, cough, and ankle pain tested positive for SARS-CoV-2 by RT-PCR and was managed with supportive care outpatient. Two weeks later, he presented to the Emergency Department with acute hypoxemic respiratory failure requiring intubation and pressor support. Inflammatory markers and BNP were markedly elevated. TTE revealed coronary artery ectasia prompting treatment with IVIG, pulse-dose steroids, anakinra, aspirin, and enoxaparin. Enoxaparin was discontinued on discharge. By two months, prednisone, anakinra, and aspirin were discontinued. The patient was asymptomatic at all follow-up appointments. Coronary arteries normalized by four months post-discharge.

Case 3

A 12-year-old Hispanic male with fever, presyncope, abdominal pain, and headaches tested positive for SARS-CoV-2 by RT-PCR and was managed with supportive care outpatient. Two weeks later, he presented after a syncopal episode and had a fever of 39.4˚C and conjunctivitis. Lab testing revealed elevated inflammatory markers. TTE found coronary artery ectasia and a small pericardial effusion. He was treated with IVIG, pulse-dose steroids, anakinra, aspirin, and enoxaparin. Enoxaparin was stopped on discharge and steroids were tapered over two months, and aspirin and anakinra were discontinued at two months. At one month post-discharge, the patient had residual weakness that resolved on subsequent follow-up. Coronary arteries normalized by two months.

Case 4

A 15-year-old black female with asthma presented with rash, myalgias, arthralgias, sore throat, and a fever of 38.3˚C. She had been exposed to a family member with COVID-19 sixteen weeks prior and tested positive for SARS-CoV-2 antibodies. She was hypotensive and hypoxic requiring pressors and supplemental oxygen. TTE showed diastolic dysfunction. She was treated with IVIG, pulse-dose steroids, anakinra, and enoxaparin. Enoxaparin was discontinued on discharge. Anakinra was stopped after one month and steroids were tapered over two months. Dyspnea on exertion and atypical chest pain persisted for one month after discharge before resolving. At three and six months follow-up, she had lingering deconditioning. Diastolic dysfunction normalized before discharge.

Case 5

A 21-year-old black male presented with rash, pharyngitis, nausea and vomiting, chest pain, and fever of 39.4˚C four weeks after testing positive for SARS-CoV-2 by RT-PCR on surveillance testing for his job. He was intubated and started on pressors. Cardiac biomarkers were elevated and MRI showed evidence of myocarditis. Inpatient treatment included IVIG, pulse-dose steroids, enoxaparin, and aspirin. Enoxaparin was stopped on discharge. Aspirin was stopped after two months and prednisone was tapered over two months. Dyspnea on exertion persisted for one month and chest pain for three months before resolving. Imaging and lab abnormalities resolved within one month.

Case 6

A 23-year-old white female presented with fever, cough, chest pain, nausea, and vomiting three weeks after testing positive for SARS-CoV-2 by RT-PCR on surveillance testing for her job. She rapidly developed acute hypoxic respiratory failure prompting intubation and pressors. She was treated with pulse-dose steroids, which were then tapered over one month. She had deconditioning at one month follow-up but was asymptomatic off medications by three months.

Case 7

A 28-year-old black male with anxiety presented with diffuse myalgias, nausea, vomiting, diarrhea, and a fever of 39˚C. He was found to be hypoxic with labs suggesting multi-organ failure in the setting of severe shock prompting intubation and pressors. Cardiac imaging was normal despite elevated biomarkers. After no response to antibiotics and negative infectious testing, he was treated with IVIG and weight-based steroids. Steroids were stopped after one week. On outpatient follow-up, he had incomplete resolution of weakness and dyspnea on exertion prompting evaluation for medical retirement from the military.

Case 8

A 41-year-old Hispanic male diagnosed with fever, cough, and shortness of breath tested positive for SARS-CoV-2 by reverse transcription-polymerase chain reaction (RT-PCR) and was managed with supportive care. Two weeks later, he returned with worsened symptoms and new chest pain. TTE revealed a moderate pericardial effusion and reduced ejection fraction of 20-25%. Cardiac biomarkers were elevated MRI suggested myocarditis. He was treated with colchicine and aspirin. Systolic function normalized before discharge. At one month follow-up, he had partial improvement in symptoms and aspirin was discontinued. He remained on colchicine and his symptoms persisted at subsequent follow-up leading to medical retirement from the military.

Patient characteristics

Pediatric patients ranged in age from 17 months to 15 years; adults were 21-41 years old. In both subsets, 50% of patients were black, 25% Hispanic, and 25% non-Hispanic white. MIS-C patients were 50% male and 50% female; MIS-A patients were 75% male and 25% female. One pediatric patient had asthma and one adult had anxiety. No other comorbidities were reported. All adults were active-duty military members.

SARS-CoV-2 exposure and MIS timing

**Figure 1:**
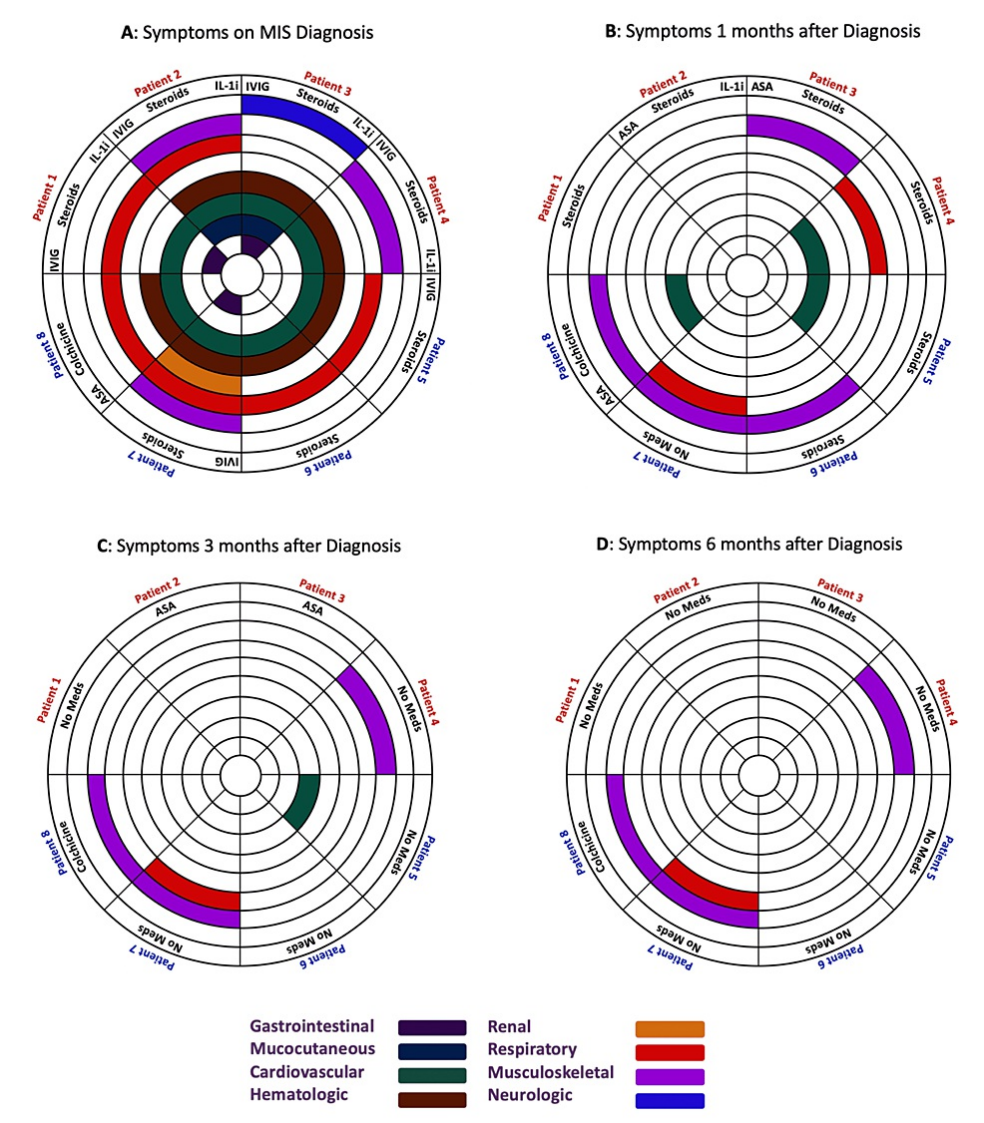
Symptoms and treatment at diagnosis and 1-, 3-, and 6-month follow-up Patients 1-4 were pediatric patients with MIS-C. Patients 5-8 were adults with MIS-A. A shows treatment and organ system involvement at the time of diagnosing MIS. B-D show treatment and organ system involvement at 1-, 3-, and 6-month follow-up, respectively. Each wedge in this circular fan chart is labeled for a patient. Each color-coded layer represents if patients had signs or symptoms of pathology in that organ system. White spaces indicate no involvement; filled colors indicate involvement per the legend at the bottom. If a patient's entire wedge is white, he or she was asymptomatic at that point in time. Medications are listed in the outermost layer. IL-1i - anakinra; ASA - aspirin; MIS - multisystem inflammatory syndrome

Table 1 details symptoms on initial exposure to SARS-CoV-2 (50% asymptomatic), time to developing MIS (1-16 weeks), and duration of hospitalization (4-18 days). Figure 1 summarizes treatment and clinical presentation at diagnosis and follow-up at one, three, and six months after discharge. Table 2 details initial and repeat laboratory results.

**Table 1:** Patient demographics, COVID-19 presentation, and MIS timing Patients 1-4 were pediatric patients with MIS-C. Patients 5-8 were adults with MIS-A. Demographics, comorbidities, home medications, COVID-19 symptoms, time from COVID-19 exposure to onset of MIS, and duration of hospitalization for MIS are listed for each patient. * - age in years, except patient 1 who was 17 months old. MIS: Multisystem inflammatory syndrome

Patient	Age*	Sex	Race	Comorbidities	COVID Presentation	Onset after COVID	Hospital Duration
1	17 months	F	Black	None	Fever, reduced feeding	1 week	11 days
2	11	M	White	None	Fever, conjunctivitis, rash, cough, arthralgias	2 weeks	18 days
3	12	M	Hispanic	None	Fever, abdominal pain, headaches	2 weeks	6 days
4	15	F	Black	Asthma	Asymptomatic	16 weeks	9 days
5	21	M	Black	None	Asymptomatic	4 weeks	4 days
6	23	F	White	None	Asymptomatic	3 weeks	16 days
7	28	M	Black	Anxiety	Asymptomatic	3 weeks	11 days
8	41	M	Hispanic	None	Fever, cough, dyspnea, chest pain	2 weeks	7 days

**Table 2:** Summary of labs Patients 1-4 were pediatric patients with MIS-C. Patients 5-8 were adults with MIS-A. ESR – erythrocyte sedimentation rate, CRP – C-reactive peptide, CBC – complete blood count, WBC – white blood cells, Hgb – hemoglobin, Hct – hematocrit, PLT – platelets, NLR – neutrophil to lymphocyte ratio, BNP – brain natriuretic peptide * - Patient 8’s repeat labs were collected 3 months after MIS-A diagnosis.

	WBC		Hgb	Hct		PLT		ESR		CRP		Ferritin		Albumin		Procalcitonin		BNP		Troponin
Units	x10^3^/mcL		g/dL	%		x10^3^/mcL		mm/hr		mg/dL		ng/mL		g/dL		ng/mL		pg/mL		ng/L
Upper Limit of Normal	9.2		16.5	50.3		407		28		0.49		307		5.2		0.25		100		20
Lower Limit of Normal	4.2		13.2	39.7		166		9		0		0		3.7		0		0		0
Patient																				
1	12.3		13.5	40.6		509		66		22.82		515		2.4		23.53		>70,000		59.2
2	1.4		12.5	38.1		233		49		25.03		2453		3.8		7.13		1330.0		522.3
3	5.0		12.1	35.7		355		41		5.45		296		4.3		---		549.8		6.13
4	13.4		12.4	37.2		265		106		23.77		1393		3.8		0.49		13,822.0		139.5
5	16.9		12.4	36.3		199		53		26.7		1611		---		8.74		9550.0		36.0
6	8.8		11.5	33.3		189		31		31.8		1335		2.6		21.33		2281.0		367.8
7	24.7		11.8	35.4		158		97		28.6		1332		2.9		---		>5000		2035.9
8	18.2		10.8	35.2		238		110		49.02		1226		3.0		6.75		4036.0		22.0
Patient																				
1	5.9		13.6	40.0		385		24		<0.3		378		5.0		---		400.0		---
2	13.4		12.4	37.2		265		106		23.77		1393		3.8		0.49		13,822.0		139.5
3	5.0		12.1	35.7		355		41		5.45		296		4.3		---		549.8		6.13
4	1.4		12.5	38.1		233		49		25.03		2453		3.8		7.13		1330.0		522.3
5	5.1		12.5	32.8		332		2		<0.3		70		4.9		---		---		<6.0
6	---		---	---		---		9		1.40		---		---		---		312.0		9.1
7	11.0		10.5	31.4		268		30		0.33		---		4.4		---		---		---
8*	4.8		13.5	39.9		213		12		0.40		1226		4.6		---		<5.0		<6.0

Cardiac involvement

All patients had cardiovascular involvement. Two children had coronary artery ectasia on echocardiography that resolved within three months. The other two children had reduced systolic function that resolved prior to discharge. Two adults had myocarditis as evidenced by chest pain, elevations in cardiac biomarkers, and meeting Lake Louise Criteria on cardiac MRI. The remaining two adults had transient reductions in the left ventricular function that resolved prior to discharge.

Hematologic involvement

Seven patients had hematologic involvement. None had thrombotic events, one had leukopenia, one had thrombocytosis, five had leukocytosis, and five had anemia. All patients had a relative lymphopenia with neutrophil-to-lymphocyte ratios ranging 3.6-56.1. These abnormalities resolved by one month in all children. One adult had lab normalization by one month, and two more showed lab normalization by three months. The fourth adult has a new, persistent anemia undergoing further evaluation at the time of submission.

Pulmonary involvement

All adults had pulmonary involvement. Three presented in acute hypoxic respiratory failure requiring intubation and inotropic support for shock. The fourth adult required supplemental oxygen but not intubation. Three adults had resolution of pulmonary symptoms by discharge. The fourth adult had persistent dyspnea on exertion at six months follow-up. Similarly, two pediatric patients presented with acute hypoxic respiratory failure requiring intubation and inotropic support for shock. Both recovered with no pulmonary symptoms at follow-up. One pediatric patient developed deconditioning while inpatient and had dyspnea and deconditioning on follow-up.

Other initial symptoms

One adult and two children had arthralgias without joint effusions that resolved after the acute phase of their illness. Similarly, one adult and two children had gastrointestinal involvement that resolved prior to discharge. The 17-month-old patient had reduced feeding, and two adults had nausea and emesis. Two children initially met incomplete Kawasaki disease criteria. One adult had acute renal failure while in shock, which resolved with volume resuscitation. One child had altered mentation and syncope with his initial presentation.

Illness severity, treatment, and outcomes

In MIS-C, two children (50%) presented with shock. All four were treated with IVIG, pulse-dose methylprednisolone, aspirin, and anakinra. Three received therapeutic-dose enoxaparin while inpatient. Glucocorticoids were tapered over eight weeks in three cases and over five weeks in one. Aspirin was continued in two patients with coronary artery ectasia and discontinued on discharge in two patients without coronary artery involvement. Anakinra was continued for four weeks in three cases and eight weeks in one. Three patients had full symptomatic resolution one month after discharge with no residual lab or radiologic abnormalities. The fourth patient had persistent deconditioning three months post-discharge. By six months, her deconditioning was persistent and she reported new depression; the other three children were asymptomatic.

In MIS-A, three adults presented with shock, two received pulse-dose methylprednisolone, one received weight-based prednisone, and two received IVIG. Two adults received aspirin for 1-2 months. Steroids were tapered over 1-8 weeks. One patient received therapeutic dose enoxaparin inpatient. One patient had residual weakness and elevated CRP one month post-discharge. Three months after diagnosis, one adult was asymptomatic while two had persistent chest pain despite normalization of lab and radiographic abnormalities. By six months, a second adult was also asymptomatic. One adult had been medically retired from the military; while asymptomatic in daily life, exercise was limited by dyspnea on exertion and exertional chest pain. The final adult was undergoing evaluation for medical retirement given persistent headaches, weakness, and fatigue. Aspirin was discontinued within four weeks in all cases of both MIS-C and MIS-A, and steroids were discontinued within eight weeks. For MIS-C, anakinra was discontinued within eight weeks for all children. All adults had residual symptoms at one month post-discharge.

## Discussion

Patient characteristics

In our MIS-C patients, 50% were male, 50% black, 25% Hispanic, and 25% white with 75% aged 11-15 years, closely matching a recent multi-center case series of 1,116 patients with MIS-C reporting 58% of patients were male, 35% black, 36% Hispanic, and 13% white with a median age of 8.9 years [11]. In MIS-A, 75% of our patients were in their 20s, 75% were male, and ethnicities were 50% black, 25% Hispanic, and 25% white. Prior literature reported the median age of MIS-A is 21 years, 70% of patients were male, and ethnicities were 36% black, 30% Hispanic, and 24% white [12].

Symptoms

Compared to the literature, our MIS-C patients had a similar number of organ systems involved (4 vs 4), similar rates of shock (50% vs 45%), and longer average hospitalization (11 vs 7 days) [10]. In MIS-A, our cohort saw less organ systems involved (3.75 vs 5), higher rates of shock (75% vs 51%), and similar hospital durations (9.5 vs 8 days) [12].

Our patients experienced a different pattern of organ system involvement compared to the literature. In MIS-C, we saw comparable rates of mucocutaneous (50% vs 44-67%) [11,13,14] and neurologic symptoms (25% vs 17-40%) [11,13], lower rates of gastrointestinal symptoms (50% vs 74-90%) [11,14,15], and higher rates of pulmonary involvement (50% vs 21%) [13]. Half of our MIS-C patients presented in shock, mirroring previous reports estimating 45-60% [11,14]. Similarly, 50% of our MIS-C cases met incomplete Kawasaki criteria compared to 22-53% of cases previously reported [15].

Comparing our MIS-A cases to a recent systematic review of the literature that included CDC surveillance data totaling 221 cases of MIS-A shows similar rates of cardiovascular involvement (100% vs 87%), lower rates of gastrointestinal (25% vs 83%), hematologic (75% vs 92%), and renal involvement (25% vs 43%), and higher rates of pulmonary involvement (100% vs 74%) in our patients [12].

Treatment

In our study, all the children received IVIG, glucocorticoids, and anakinra, whereas no adult received this combination. For comparison, MIS-C studies report 71-76% of patients received IVIG, 52-55% glucocorticoids, and 7-8% IL-1 inhibitors [11,15] with 13% receiving no immunomodulatory medication [11]. In MIS-A, 74% of patients were treated with glucocorticoids, 55% with IVIG, and 21% with immunomodulatory medications [12].

We hypothesize that the difference in treatment between pediatric patients and adults reflects that working criteria for MIS-A were published several months after those for MIS-C, which could account for both lesser recognition of the disease and lack of clarity in MIS-A treatment approaches. In fact, three out of four of our adult cases were transferred from other hospitals, and referring physicians in these cases pondered if MIS-C criteria and protocols were generalizable to adults. Similarly, the authors from the Swedish MIS-A case series acknowledge they used MIS-C treatment algorithms to guide their MIS-A care [10]. Further, the aforementioned systematic review on MIS-A highlighted that over 50% of cases of MIS-A were incorrectly reported in the MIS-C database [12] suggesting that adult physicians use pediatric resources to guide MIS-A management, which is concordant with the joint CDC and IDSA MIS-A resource center referencing MIS-C guidelines [8].

Outcomes

Among our patients, children had better outcomes than adults, raising the question of how much of the difference is due to physiologic variance in children and adults versus how much is attributable to treatment differences. Turning to the literature, a cohort of 46 patients with MIS-C in the UK where 83% of patients received IVIG, 54% methylprednisolone, and 13% anakinra, found that 37% of patients were asymptomatic, 39% had persistent neurologic symptoms, 15% psychologic symptoms, 13% gastrointestinal symptoms, and 9% had weakness six months after diagnosis [9]. A small case series of four adults with MIS-A in Sweden reported that 100% received methylprednisolone and 75% received IVIG and anakinra [10]. The authors report “no severe complications” at three months follow-up but did not report on presence or absence of symptoms [10].

Limitations

This study has several limitations. First, this was a small retrospective study. Accordingly, some data of interest was not available and treatment patterns do not necessarily represent the current standard-of-care as treatment recommendations have changed and, in some cases, were not available on initial presentation. The small sample size of our cohort and practice within the military health system may limit generalizability as costs are lower and access to care is higher. Additionally, the adults in this cohort were active-duty military members. To serve, they passed a medical screening exam and continue to undergo a medical evaluation for fitness-for-duty annually, selecting for a healthy population and limiting generalization to older adults or those with comorbidities.

## Conclusions

MIS appears to be monophasic as evidenced by all of our patients discontinuing glucocorticoids and immunomodulatory medications within two months without recurrence. We saw better outcomes in children than in adults. Children were more likely to be symptomatic after initial exposure to SARS-CoV-2 leading to earlier detection of MIS and likely influencing the greater use of combination therapy with glucocorticoids, IVIG, and anakinra in MIS-C than in MIS-A. How much of the better outcomes are attributable to earlier detection and more aggressive therapy versus differences in pediatric and adult physiology remains unclear. Given the similarity in the presentation of MIS-C and MIS-A, and success in extrapolating MIS-C guidelines to adults, we suspect the former. We acknowledge that further study investigating these differences and comparing the efficacy of early combination therapy to step therapy would be insightful.
